# ChatGPT can assist physicians in managing Parkinson’s disease and resolving DBS hesitancy

**DOI:** 10.1097/JS9.0000000000001996

**Published:** 2024-07-25

**Authors:** Hongjin Wang, Dan Shan, Yujiao Wu

**Affiliations:** a Department of Cardiovascular Surgery, Longyan First Affiliated Hospital of Fujian Medical University, Longyan; b Department of Pharmacy, Changzhou Third People’s Hospital, Changzhou Medical Center, Nanjing Medical University, Nanjing, China; c Department of Biobehavioral Sciences, Columbia University, New York, NY, USA

*Dear Editor,*

In 1987, Benabid utilized deep brain stimulation (DBS) of the ventrolateral thalamic nucleus to effectively treat Parkinson’s tremor^1^. Since then, DBS has been used to treat muscular dystonia, including essential tremor and Parkinson’s disease (PD), in numerous institutions across the world. In recent years, the concepts, techniques, and approaches to treating PD have been constantly updated, and DBS has become an important non-drug treatment for PD due to its effectiveness and safety.

We read with great interest Meng *et al.*’s^2^ article titled ‘Utilization, surgical populations, centers, coverages, regional balance, and their influential factors of deep brain stimulation for Parkinson’s disease: a large-scale multicenter cross-sectional study from 1997 to 2021’, which was recently published in the *International Journal of Surgery*. They concluded that despite the rapid improvement of the surgical population in China, the coverage of DBS for PD is still lower than that of foreign countries and is insufficient to cover potentially eligible patients. Due to economic and medical resource disparities between regions, as well as concerns among patients about surgical effectiveness, invasiveness, cost, and irreversibility, the use of DBS has been greatly limited. One study showed that even among patients who were prepared to undergo DBS surgery, more than 45% belonged to the reluctant group^3^. DBS hesitation is so common that it seems difficult for physicians to solve this problem.

In the past year, large language models represented by ChatGPT have received a lot of attention. ChatGPT has been hailed as a disruptive technology and has shown potential for application in multiple medical fields^4^. For doctors, using ChatGPT can generate text for patient education. For example, ChatGPT could explain why patients using deep brain stimulators should not go through metal detectors. This extraordinary ability can reduce physician fatigue and time management, ultimately benefiting patients.

For patients, the main reason for DBS hesitancy is fear of complications and financial burden. Patients can present their concerns to ChatGPT and ChatGPT will provide smooth, personalized and comprehensive answers. Completed studies have shown that ChatGPT has a higher proportion of responses rated as good or very good quality than physicians, and the responses have also been rated as more empathetic than physician responses^5^. However, accountability cannot be avoided. There must be someone responsible for the responses provided by ChatGPT, which helps ensure the responsible and ethical utilization of artificial intelligence technology by physicians.

Undoubtedly, ChatGPT still has certain limitations. Although ChatGPT can provide targeted answers to the questions raised by patients, it depends on general knowledge and shared perspectives rather than specific patient situations. The value of a physician’s expertise, derived from physical examinations and in-person conversations, is irreplaceable in tailoring responses to individual patient needs. It is worth noting that ChatGPT’s answers cannot be used as medical advice, as this may be a risk during the use of ChatGPT. Recently, there have been reports that some institutions have announced a ban on the use of ChatGPT at academic and social events for safety and ethical reasons. This action is one-sided. The use of ChatGPT should be restricted rather than completely negated. At least, as we have said, ChatGPT has shown great potential in helping doctors treat Parkinson’s disease and resolve DBS hesitancy. Laws and regulations should be improved to help people make better use of ChatGPT.

## Ethical approval

Not applicable.

## Consent

Not applicable.

## Source of funding

Not applicable.

## Author contribution

H.W. : manuscript writing; D.S. and Y.W. : study design and manuscript revision.

## Conflicts of interest disclosure

The authors declare no conflicts of interest.

## Research registration unique identifying number (UIN)

Not applicable.

## Guarantor

Yujiao Wu.

## Data availability statement

Not applicable.

## Provenance and peer review

Not applicable.
